# Supporting prescribing in Irish primary care: protocol for a non-randomised pilot study of a general practice pharmacist (GPP) intervention to optimise prescribing in primary care

**DOI:** 10.1186/s40814-018-0311-7

**Published:** 2018-07-05

**Authors:** Karen Cardwell, B. Clyne, F. Moriarty, E. Wallace, T. Fahey, F. Boland, L. McCullagh, S. Clarke, K. Finnigan, M. Daly, M. Barry, S. M. Smith, on behalf of the General Practice Pharmacist (GPP) Study Group, Catriona Bradley, Paul Gallagher, Ciara Kirke, Edel Murphy, Andrew Murphy, Patrick Byrne, Aisling Croke

**Affiliations:** 1 Health Research Board Centre for Primary Care Research, Royal College of Surgeons in Ireland, 123 St Stephen’s Green, Dublin, Ireland; 2 Department of Pharmacology and Therapeutics, Trinity College Dublin, Dublin, Ireland; 3 Health Service Executive Medicines Management Programme, Dublin, Ireland

## Abstract

**Background:**

Prescribing for patients taking multiple medicines (i.e. polypharmacy) is challenging for general practitioners (GPs). Limited evidence suggests that the integration of pharmacists into the general practice team could improve the management of these patients. The aim of this study is to develop and test an intervention involving pharmacists, working within GP practices, to optimise prescribing in Ireland, which has a mixed public and private primary healthcare system.

**Methods:**

This non-randomised pilot study will use a mixed-methods approach. Four general practices will be purposively sampled and recruited. A pharmacist will join the practice team for 6 months. They will participate in the management of repeat prescribing and undertake medication reviews (which will address high-risk prescribing and potentially inappropriate prescribing, deprescribing and cost-effective and generic prescribing) with adult patients. Pharmacists will also provide prescribing advice regarding the use of preferred drugs, undertake clinical audits, join practice team meetings and facilitate practice-based education. Throughout the 6-month intervention period, anonymised practice-level medication (e.g. medication changes) and cost data will be collected. A nested Patient Reported Outcome Measure (PROM) study will be undertaken during months 4 and 5 of the 6-month intervention period to explore the impact of the intervention in older adults (aged ≥ 65 years). For this, a sub-set of 50 patients aged ≥ 65 years with significant polypharmacy (≥ 10 repeat medicines) will be recruited from each practice and invited to a medication review with the pharmacist. PROMs and healthcare utilisation data will be collected using patient questionnaires, and a 6-week follow-up review conducted. Acceptability of the intervention will be explored using pre- and post-intervention semi-structured interviews with key stakeholders. Quantitative and qualitative data analysis will be undertaken and an economic evaluation conducted.

**Discussion:**

This non-randomised pilot study will provide evidence regarding the feasibility and potential effectiveness of general practice-based pharmacists in Ireland and provide data on whether a randomised controlled trial of this intervention is indicated. It will also provide a deeper understanding as to how a pharmacist working as part of the general practice team will affect organisational processes and professional relationships in a mixed public and private primary healthcare system.

## Background

Prescribing in the context of polypharmacy (i.e. multiple medicines) is complicated, as the potential for drug-drug interactions, adverse drug reactions (ADRs) and potentially inappropriate prescribing (PIP) is increased [1]. PIP is defined as the prescribing of medicines where the risk of an adverse event outweighs the clinical benefit or the omission of medicines which are clinically indicated. PIP has been associated with outcomes such as increased hospital admissions [2], accident and emergency (A&E) visits [3], ADRs [4] and healthcare costs [5, 6]. The management of patients with multimorbidity who are taking multiple medicines is challenging, particularly for GPs in primary care, as there is often fragmentation, or poor communication, between primary and secondary care settings [7]. Thus, there has been an increased emphasis on the need to support GPs in the management of these patients [8]. One approach has been the integration of pharmacists into the general practice team. This co-location of pharmacists in general practices has positively affected various areas of chronic disease management and the quality use of medicines [9].

In 2015, the United Kingdom (UK) National Health Service (NHS), a publically funded healthcare system, introduced a pilot practice-based pharmacist initiative in response to a workforce action plan for general practice [10]. To date, studies have shown that pharmacists, working as part of the general practice team, have influenced the safety and quality of prescribing, which has the potential to improve patients’ outcomes [9]. However, there is limited evidence on the impact of a practice-based pharmacist on GP workloads and patient outcomes [11], and it is still unclear whether such interventions are cost-effective, particularly in relation to other approaches to medicines management such as utilisation of electronic prescribing systems and education [8, 12, 13].

Unlike the UK, pharmacists in Ireland have not been integrated into the general practice team. Thus, the feasibility of the integration of pharmacists into Irish general practice teams warrants exploration, prior to evaluation in a full-scale randomised controlled trial (RCT). Before proceeding to a definitive RCT, the intervention will be further refined based on the results of this current study and evolving research literature in this area. The aim of this pilot study is to develop and test an intervention (defined as the general practice pharmacist [GPP] intervention) involving pharmacists working with GPs to optimise prescribing in Ireland, which has a mixed public and private primary healthcare system. The study will determine the costs and potential effectiveness of the GPP intervention and, through engagement with key stakeholders, will explore the potential for an RCT of the GPP intervention in Irish general practice settings.

## Methods

### Study design

This will be a non-randomised pilot study. Pilot studies are used to determine if and how an intervention can be delivered in practice. Pilot studies implement (or part-implement) an intervention on a smaller scale to help researchers decide how to proceed with that intervention in a future, larger study [14]. Non-randomised pilot studies are studies in which all or part of the intervention to be evaluated, and other processes to be undertaken in a future trial, are carried out (piloted) but without randomisation of participants [14]. A qualitative process evaluation and cost of care analysis of the GPP intervention will be conducted. The study will last for 24 months and will be reported using the principles of the Consolidated Standards of Reporting Trials guidelines extension for the reporting of randomised pilot and feasibility studies [15]. The main study intervention period (i.e. the time during which pharmacists will be working within the general practice setting) will last for 6 months. Throughout this time, participating practices will be asked to display a notice informing patients of the study and the role of the pharmacist within the practice team during the intervention period. Should a patient wish to opt-out of the study they can indicate this to the practice staff and subsequently the pharmacist will not access their records. During months 4 and 5 of the 6-month intervention period, a nested Patient Reported Outcome Measures (PROM) study will be undertaken to permit the collection of individual patient data. The nested PROM study was scheduled to take place during months 4 and 5 of the 6-month intervention period as we hypothesised that this would give time for working practices to stabilise and allow pharmacists and the general practice team to familiarise themselves with the implementation of the intervention components at the practice.

### Main study population and recruitment

The study will be conducted in four general practices. Practices eligible for invitation to the study will be identified from the Health Research Board (HRB) Centre for Primary Care Research (CPCR) in Ireland research network. Practices will be eligible to participate if they have ≥ 1000 older patients (aged ≥ 65 years) on their patient panel. This will ensure adequate numbers of patients with significant polypharmacy (defined here as ≥ 10 repeat medicines) and allow the feasibility of the intervention to be tested in the nested PROM study. Practices will be purposively selected to reflect a range of practice sizes and types, from both socioeconomically deprived and affluent areas. Eligible practices will be invited to participate via a letter of invitation sent to the practice by email. The invitation email will include a study information pack which consists of a study information leaflet outlining the steps of the intervention and availability of continuing medical education points for participation in the study, practice profile questionnaire and practice consent form.

### Nested PROM study population and recruitment

Using a tool embedded within the general practice prescribing software, the pharmacist will compile a list of patients with significant polypharmacy (defined as ≥ 10 repeat medicines). A random sample of these patients will be selected by the pharmacist and screened for inclusion in the nested PROM study. Patients will be eligible for inclusion in the nested PROM study if they are aged ≥ 65 years and are able to attend their practice or participate in data collection. Patients will be excluded if they have psychiatric or psychological morbidity or cognitive impairment sufficient to impair the provision of informed consent, if they have a life-limiting illness likely to lead to death or major disability during the study follow-up period, or if they have already had a medication review/interacted with the pharmacist during the study period.

**Fig. 1:**
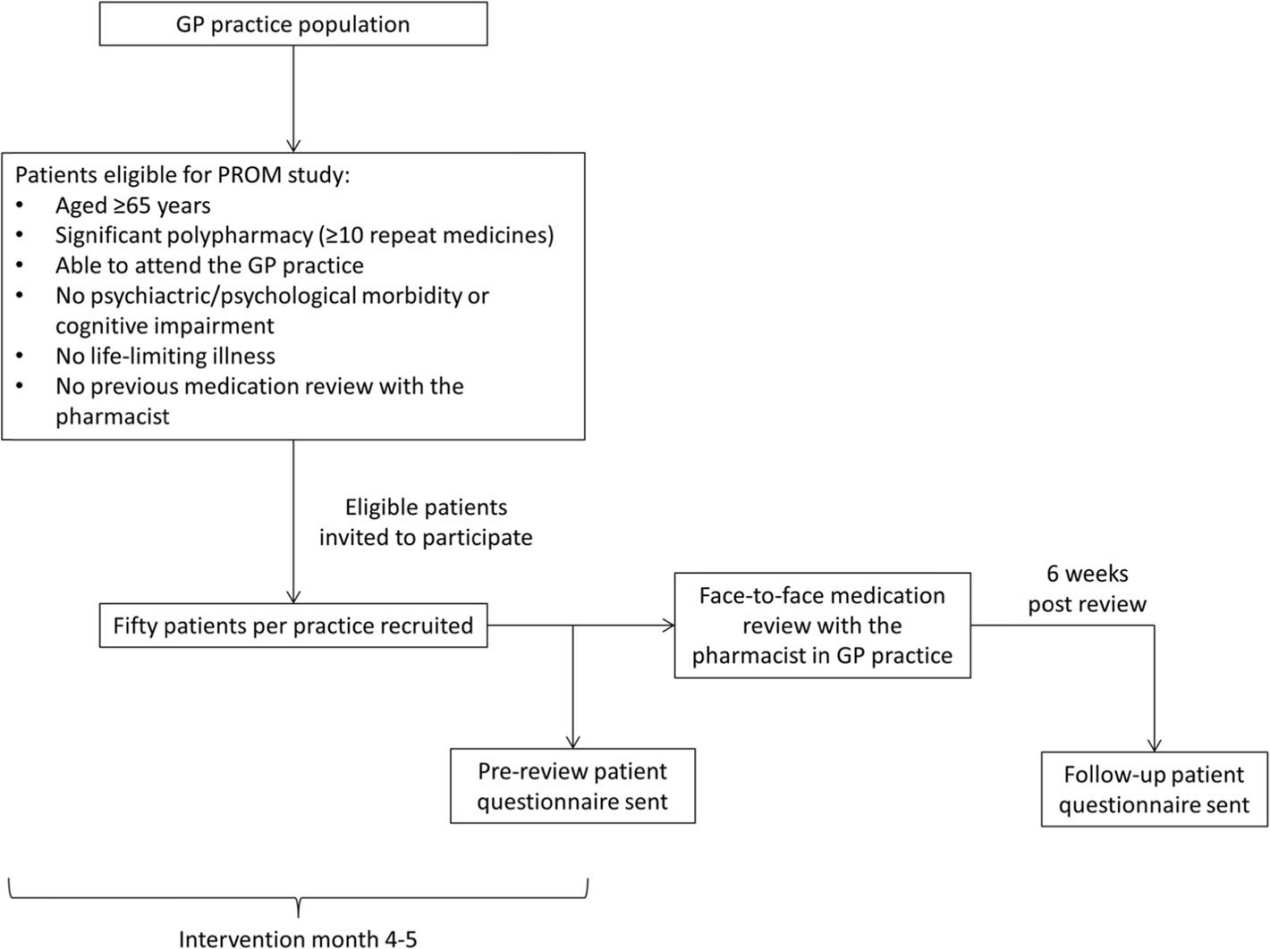
Figure showing the flow of patients through the study

A letter of invitation (printed on practice headed paper) and a patient information pack will be posted to eligible patients. The patient information pack will include a study information leaflet, consent form and questionnaire to explore their perceived level of health. The letter of invitation will invite them to attend an appointment with the pharmacist for a medication review; they will also be asked to bring their completed questionnaire to this appointment. Eligible patients will be invited until a total of 200 patients (50 patients from each practice) are recruited. Written informed consent will be obtained from these patients (by the pharmacist) prior to any data collection, and follow-up questionnaires will be sent to these patients 6 weeks after the pharmacist has left the practice. Figure 1 gives an overview of the flow of patients through the study.

### Intervention development

The design and development of the GPP intervention was informed by the initial stages of the Medical Research Council (MRC) Framework for the design and evaluation of complex interventions to improve health [16]. Within this framework, the MRC defines four stages (development, feasibility and piloting, evaluation and implementation) through which complex interventions should be developed and evaluated. This process is described as ‘fluid’ in nature as researchers may move back and forward between stages, depending on process outcomes and emerging evidence.

The MRC recommends that the ‘development’ of complex interventions be systematic using the best available evidence [16]. The GPP intervention builds upon previous and ongoing research in the Health Research Board (HRB) Centre for Primary Care Research (CPCR) in Ireland, which has focused on PIP and polypharmacy. This includes the OPTI-SCRIPT trial [17], a multifaceted intervention to decrease PIP, and the SPPiRE study [18], the definitive RCT of an adapted version of the OPTI-SCRIPT intervention. Additionally, previous evaluation reports and guidance documents published by the Health Service Executive (HSE) Medicine Management Programme (MMP) have informed the development of this intervention. The HSE is responsible for the management of public health services in Ireland, and the MMP is a multidisciplinary National Clinical Programme with a focus on the safe, effective and cost-effective use of medicines. The MMP aims to provide national leadership on the quality of medicines management, access to medicines and overall expenditure on medicines [19].

### Proposed intervention components

**Fig. 2:**
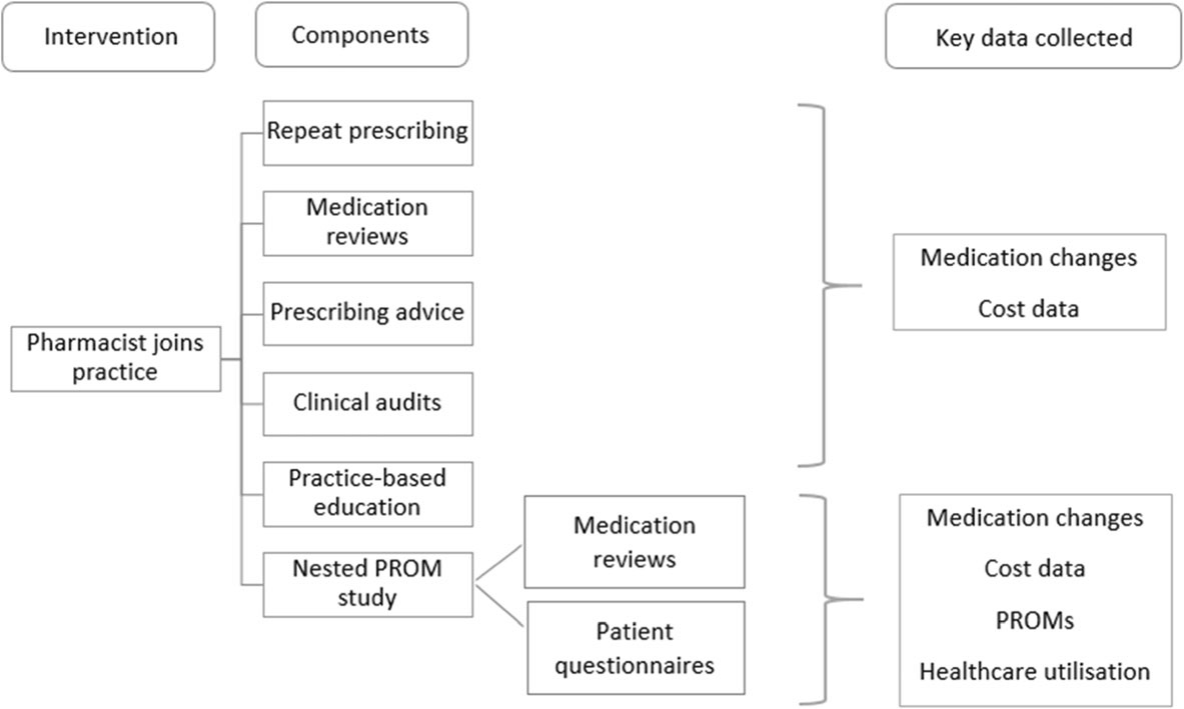
Overview of the general practice pharmacist intervention and its components

One pharmacist will join the general practice team for a period of 6 months, in accordance with a letter of agreement between them and the practice. The pharmacists will be experienced community-based clinical pharmacists by background and training. The pharmacist will participate in the routine management of practice repeat prescribing (i.e. clinically review repeat prescribing requests made by patients and highlight any issues to the prescriber) and conduct medication reviews (opportunistic and targeted). These medication reviews will focus on high-risk prescribing practices, defined by Guthrie et al. as the prescribing of medication(s) whereby the risk outweighs the benefit [20], PIP as defined by the modified STOPP/START criteria used in the OPTI-SCRIPT trial [17], and SPPiRE study [18], assessment of need for commonly used preventive drugs according to clinical guidelines and the deprescribing of medication(s) that may cause harm or are no longer providing benefit [21]. Pharmacists will also provide prescribing advice and promote recommendations from the MMP relating to cost-effective and generic prescribing [22], as well as promotion of the preferred drug initiative (which promotes the prescribing of a ‘preferred drug’ within an number of specific drug classes) [23]. Additionally, pharmacists will join routine clinical practice meetings and, if appropriate, be available to conduct education sessions based on the specific needs of the practice and support GPs in undertaking clinical audits that can support the GPs’ annual Continuing Professional Development requirements. In the first instance, medication reviews will be chart-based, followed by a patient-facing review as deemed appropriate (this will be determined by the GP). In Ireland, pharmacists cannot prescribe, and therefore, the GP will maintain clinical autonomy throughout the intervention period and all decisions will be verified by the GP prior to any action being taken. Finally, during months 4 and 5 of the study intervention period, pharmacists will recruit 50 patients per practice (total 200 patients) to the nested PROM study. These patients will be invited to a face-to-face medication review with the pharmacist and asked to complete a patient questionnaire to permit the collection of PROMs. Figure 2 provides an overview of the GPP intervention and its components.

### Comparison group

This will be an uncontrolled pilot study, so there will be no formal comparison group. However, comparison data will be available from the control group of the parallel SPPiRE trial [18], an on-going RCT of a complex intervention to support medicines management for patients ≥ 65 years with multimorbidity in Irish primary care. SPPiRE will also use HSE Primary Care Reimbursement Services data, the national database for pharmacy claims. Data from this national prescribing database will be used as a contemporary national control to compare study practices and non-participating practices and determine if there have been changes in outcomes over the study period at the population-level.

### Outcome measures

As this is a pilot study, a range of medication-related outcome measures will be collected to explore their potential use in an RCT. These outcomes will be collected at practice-level throughout the 6-month intervention period (i.e. main study data) and patient-level during months 4 and 5 of the intervention period (i.e. nested PROM study data). See Table 1 for a summary of the outcomes measured in this study. It provides a comprehensive overview of these outcomes, along with a definition for each outcome collected, and a description of the data source from which the outcome will be recorded.

**Table 1:** Summary of the outcomes collected during the General Practice Pharmacist intervention

	Definition	Data Sources & Measurement
Main Study Outcomes (anonymised practice-level data collected throughout six-month intervention)		
Demographics		
Patient age	Age in years	Patient health record
Gender	Male, Female, Other	Patient health record
Patient status	Eligibility for free general practice care	Patient health record
Primary Outcomes		
Patients eligible for review	Number and proportion of patients with polypharmacy presenting for repeat prescriptions per day	Patient health record Pharmacist data collection tool
Medicines reviews	Number of reviews undertaken	Pharmacist data collection tool
Repeat medications	Number and proportion of repeat medications currently prescribed per patient reviewed	Patient health record Pharmacist data collection tool
Polypharmacy	Number and proportion of patients (who request a repeat prescription during the intervention period) with polypharmacy (≥10 repeat medicines)	Patient health record Pharmacist data collection tool
Medicines stopped, started and dose alterations	Number and proportion of medicines stopped, started and altered per patient reviewed	Pharmacist data collection tool
High risk prescribing and potentially inappropriate prescribing	Number and proportion of instances considered to be high risk or potentially inappropriate	Patient health record • High risk prescribing indicators • Modified STOPP/START criteria from OPTI-SCRIPT and SPPiRE
Deprescribing	Number and proportion of episodes of deprescribing per patient reviewed	Pharmacist data collection tool
Generic prescribing	Proportion of generic prescribing per practitioner	Pharmacist data collection tool
Use of Medicines Management Programme preferred drug and prescribing and cost guidance recommendations	Number and proportion of patients with Medicines Management Programme preferred drug per item prescribed per patient reviewed	Pharmacist data collection tool
Adverse drug reactions	Number of adverse drug reactions that caused withdrawal of prescribed medication(s)	Patient health record Pharmacist data collection tool
Inadequate prescription instructions	Number of prescriptions with inadequate instructions (e.g. ‘as directed’)	Patient health record Pharmacist data collection tool
Specific instances of high risk prescribing and potentially inappropriate prescribing	Number and proportion of the following instances: • Long-term use (i.e. >3 months) of non-steroidal anti‐inflammatory drug • Therapeutic duplication • Long terms use (i.e. >3 months) of corticosteroids (e.g. prednisolone) without bisphosphonate	Patient health record Pharmacist data collection tool
Nested PROM Study Outcomes (patient-level data collected during months four and five, pseudo-anonymised until follow-up complete)		
Demographics		
Patient age	Age in years	Self-report Patient health record
Gender	Male, Female, Other	Self-report Patient health record
Marital status	Current marital status at time of baseline questionnaire	Self-report
Ethnicity	Country in which patient was born	Self-report
Education	Highest level of education achieved at time of baseline questionnaire	Self-report
Socioeconomic status	Eligibility for free general practice care and deprivation level of electoral division wherein patient resides and their occupation	Eligibility for free general practice care will be based on self-report and confirmed by patient health record. Deprivation level of electoral division wherein patient resides classified according to Small Area Health Research Unit National Deprivation index. Occupation will be coded with Central Statistics Office occupational categories.
Health related Quality of life [33]	Health related Quality of life five level questionnaire	Self-report
Patients' attitudes towards deprescribing [34]	Patients’ Attitudes Towards Deprescribing questionnaire	Self-report
Treatment burden (unpublished)	Treatment Burden Questionnaire	Self-report
Beliefs about Medicines Questionnaire [35]	Beliefs about Medicines Questionnaire	Self-report
Number of repeat medications	Number of repeat medications currently prescribed	Patient health record
High risk prescribing and potentially inappropriate prescribing	Number and proportion of instances considered to be high risk or potentially inappropriate	Patient health record • High risk prescribing indicators • Modified STOPP/START criteria from OPTI-SCRIPT and SPPiRE
Healthcare utilization		
GP Visits	Number of GP visits	Patient health record
A&E visits	Number of A&E visits	Patient health record Self-report
Hospital admissions (emergency and elective)	Number and rate of admissions Length of stay in days (if admitted) Time to first admission and time to re-admission (days)	Patient health record Self-report
Practice nurse visits	Number of practice nurse visits	Patient health record Self-report
Other primary care visits	Number of visits to other primary care services and identification of those services	Patient health record Self-report
Out-patient department visits	Number of visits to out-patient department services	Patient health records Self-report
Cost Data		
Healthcare utilization	As outlined in the ‘Healthcare utilisation’ section above	Patient health records Self-report
Patient costs	Travel Occupation	Self-report
Staff costs	Costs related to the recruitment and training of the pharmacist Practice-based education of GPs and associated staff also taken into account	Costs per hour (for GP/nurse/secretary input) and per day for pharmacist (use Health Service Executive pay grades for each grade of staff)
Medication costs	Price for drugs supplied through the community drug schemes are listed in the reimbursement files of the Primary Care Reimbursement Service	Primary Care Reimbursement Service reimbursement files
Process Evaluation Data		
Key stakeholder opinions	Stakeholder perspectives	Qualitative interview transcripts

### Sample size

As this is a pilot study, a formal sample size calculation will not be required [24]. The aim is to recruit four practices and, for the nested PROM study, 50 patients per practice. This will be a large enough sample to inform about the practicalities of delivering the intervention, general practice uptake and recruitment and retention of pharmacists.

### Main study data collection and analysis

Throughout the 6-month intervention period, quantitative practice-level data will be collected by the pharmacists using the predefined data collection tool (see Table 1 for more details). These data will be aggregated at the practice-level and will not contain any patient identifiers. Descriptive statistics and estimation using confidence intervals will be the main focus of the analysis. For categorical measures, frequencies and percentages will be presented, and for continuous measures, the mean and standard deviation (SD) will be reported. For continuous measures which show evidence of some skew, a median and interquartile range may also be presented or substituted for the mean and SD.

### Nested PROM study data collection and analysis

When the intervention has been running and processes have been standardised for that practice, PROM data will be collected (following provision of written informed consent). This will be collected during months 4 and 5 of the intervention period in each practice. Eligible patients will be invited to attend a medication review with the pharmacist, and PROM data will be collected using the patient questionnaire (to be completed pre-intervention, i.e. before the medication review with the pharmacist). A follow-up patient postal questionnaire will also be completed. This follow-up questionnaire will be conducted 6 weeks after the medication review with the pharmacist (i.e. post-intervention). Reminders will be sent to any patients who have not completed the follow-up questionnaires; these reminders will be posted 1 month after the follow-up questionnaire has been posted. Demographic and clinical characteristics of patients will be presented using appropriate descriptive statistics (e.g. proportions, means). Differences in outcomes pre- and post-intervention will also be explored. For categorical data, McNemar’s test will be used and a paired-samples *t* test or Wilcoxon signed-rank test (depending on normality of the data) will be used for continuous data. Data analysis will be conducted using Stata and *p* values < 0.05 will be deemed significant.

### Qualitative process evaluation

Qualitative data will be collected using semi-structured interviews which will be based on a predefined interview topic guide. Pre- and post-intervention interviews will be conducted (by KC and BC) with four GPs, three pharmacists, four nurses and four practice managers from participating GP practices, as well as with eight patients who participated in the nested PROM study. Pre-intervention interviews will be conducted at least 1 week before the intervention commences, and post-intervention interviews will be conducted 6 weeks after the intervention has taken place. Interviews will last approximately 30–60 min, and the topic guide will explore issues related to context, fidelity and implementation of the intervention, as well as the experiences of those participating. Interviews will be conducted either in person or via telephone. Telephone interviewing is generally used where time or budget is limited, and evidence suggests there is little difference in answers obtained in person or via telephone [25]. All interviews will be audio recorded (on loudspeaker for telephone interviews).

All interviews will be transcribed verbatim, and all participant data will be pseudo-anonymised by assignment of a unique study identifier (until completion of follow-up interviews, thereafter all interview data will be completely anonymised). A thematic analysis will be conducted following a six-step process and employing Normalisation Process Theory (NPT) [26] to understand how the intervention was (or was not) embedded in routine clinical practice, and relational coordination [27] to explore interprofessional collaboration. NPT is concerned with understanding the dynamics of implementing, embedding, and integrating a complex intervention within a healthcare system. NPT has four components which can be used to evaluate implementation of complex interventions, these components are coherence, cognitive participation, collective action and reflexive monitoring. Coherence encompasses whether the intervention makes sense to, and is perceived to be of value to, the relevant participants, and whether it fits with the goals and activities of the organisation. Cognitive participation considers whether participants will be prepared to invest in the new intervention. Collective action asks what effect the intervention will have on current work and whether it is consistent with the existing practices. Finally, reflexive monitoring asks how participants perceive the intervention once it has been in place for a while [26]. Contextual factors and potential barriers relating to the mixed public and private funding of Irish Primary Care will also be explored. Researchers will independently review the transcripts of the individual interviews several times to familiarise themselves with the relevant data. Small sections of data will be assigned a code that summarises the content. Codes with common features will be grouped together in emerging themes, before finally being assigned to overarching themes [28]. In the write-up, quotations will be used as exemplars of key themes. NVivo 10® will be used to index and organise the data for analysis. In addition, demographic characteristics of the pharmacists and GPs involved will be displayed and taken into account in the analysis process. Moreover, the potential timesaving (or time-intensification) aspects of the intervention will also be explored during the process evaluation. However, given the small sample size, this cannot be accurately determined.

### Economic analysis

A cost of care study will be conducted alongside the pilot study to determine the direct costs and related cost savings of the proposed intervention. Comparator cost data will be available from the control group of the parallel SPPiRE trial [18] and the national PCRS prescribing reimbursement data. The completion of a costing study will allow us to consider potential cost-effectiveness and will guide a future cost-effective analysis (as a secondary outcome measure), if an RCT is carried out. The costing study will be based on the cost of providing the intervention and the cost savings realised from the intervention. Costs related to the recruitment and training of the pharmacists, practiced-based education of the GPs and associated staff will also be accounted for. Data used in the economic analysis will include:

- Cost of changes to prescriptions to address prescribing that is high risk or potentially inappropriate, as documented by the pharmacist;
- Cost of deprescribing by calculating original cost of prescriptions and final cost due to changes in the items prescribed;
- Cost savings on the number of items changed from branded to generic prescribing;
- Cost savings on the number of preferred drugs which have been switched from other agents;
- Cost savings following assessment of requirement for commonly used preventive drugs such as aspirin and cholesterol-lowering agents;
- Resources used in the reviewing of nursing (residential) home patients and changes to prescriptions will be measured and costed;
- Reduced wastage of medicines due to rationalising polypharmacy will be measured and the associated cost ascertained.

### Continuation criteria

Continuation criteria (see Table 2) will be considered to determine whether further evaluation of this intervention is warranted (to test the effectiveness of the intervention). Continuation criteria (also referred to as progression criteria) are based on consideration of the primary objectives around feasibility and the potential for effectiveness and system-wide implementation. Quantitative and qualitative process evaluation data will be analysed to consider the following continuation criteria:

- Successful general practice uptake and recruitment and retention of pharmacists;
- Successful implementation of the intervention and consideration of treatment fidelity criteria [29];
- Process evaluation indicates that the intervention is acceptable to general practice teams and patients;
- Positive effect on medicines-related outcomes;
- Cost of care study indicates that the intervention might be cost saving.

**Table 2:** Continuation criteria which indicate whether to proceed with a randomised controlled trial

Proceed with RCT	Proceed with RCT following some changes to the protocol	Do not proceed with RCT unless problems can be overcome
Recruitment of 4 general practices within 3 months	Recruitment of 4 general practices within 3–6 months	Unable to recruit 4 general practices within 6 months
Retention of ≥ 3 general practices throughout 6-month intervention period	Retention of 2 general practices throughout 6-month intervention period	Retention of < 2 general practices throughout 6-month intervention period
Recruitment of 3 pharmacists within 3 months	Recruitment of 3 pharmacists within 3–6 months	Unable to recruit 3 pharmacists within 6 months
Retention of ≥ 2 pharmacists throughout 6-month intervention period	Retention of 1 pharmacist throughout 6-month intervention period	Retention of 0 pharmacists throughout 6-month intervention period
Recruitment of 50 PROM study patients from each practice	Recruitment of 25–49 PROM study patients from each practice	Recruitment of < 25 PROM study patients from each practice
Retention of ≥ 35 PROM study patients for follow-up	Retention of 25–34 PROM study patients for follow-up	Retention of < 25 PROM study patients for follow-up
GPP intervention acceptable to ≥ 75% GPs, pharmacists and patients involved	GPP intervention acceptable to 50–74% GPs, pharmacists and patients involved	GPP intervention acceptable to < 50% GPs, pharmacists and patients involved
Delivery of GPP intervention feasible	Delivery of GPP intervention partially feasible	Delivery of GPP intervention not feasible
GPP intervention demonstrates cost savings which outweigh the cost of implementing the intervention	GPP intervention demonstrates potential for cost savings which outweigh the cost of implementing the intervention	GPP intervention does not demonstrate potential cost savings which outweigh the cost of implementing the intervention

*GP* general practitioners, *GPP* general practice pharmacist, *PROM* Patient Reported Outcome Measures, *RCT* randomised controlled trial

### Data management and protection

A data management plan will be developed and agreed by the study steering committee. Practice-level data will be collected from patient health records and will be completely anonymised. PROM data will be collected using patient health records, completed questionnaires and self-reporting (during the medication review). These data will be pseudo-anonymised since contact details will be required for patient follow-up and interview (with patient consent). A unique study identifier number will be given to each PROM participant (this will be recorded in the data collection tool for baseline PROM data collection). In a separate document, the study identifier number and participant’s contact information will be recorded. These data will be used for study follow-up purposes only and will only be accessed by the pharmacist at that practice. No PROM data will leave the practice during the intervention period. Upon completion of the follow-up with each PROM participant, PROM study data will also be completely anonymised. Thereafter, it will leave the practice for analysis.

Qualitative evaluation data will be collected via pre- and post-intervention semi-structured interviews which will be audio recorded and transcribed verbatim. Similarly, data collected during the pre-intervention interview will be pseudo-anonymised until the post-intervention follow-up interviews have been conducted. Thereafter, all interview data will be completely anonymised.

All practice and PROM data will be stored electronically on secure password-protected hard drives and transferred to a secure password-protected server. Hard copies of data (i.e. completed questionnaires and consent forms) will be scanned and stored electronically on a secure password-protected server; the original copies will be shredded. Completed, anonymised questionnaires will only leave the practice on completion of follow-ups. Audio-recordings will be transcribed and stored electronically and the original recording deleted; all transcripts will be completely anonymised. The principles of FAIR data will be applied when considering outcomes for follow-up studies in this area.

### Ethical approval

Ethical approval was granted by the Research Ethics Committee of the Irish College of General Practitioners.

## Discussion

This study aims to test an intervention (GPP intervention) involving pharmacists, working within GP practices, to optimise prescribing in a mixed public and private primary healthcare system in Ireland. The intervention will focus on patients aged ≥ 65 years with significant polypharmacy (≥ 10 repeat medicines) and will utilise a number of methodologies including the review of community-based and nursing (residential) home patients for instances of prescribing practices considered to be high risk or potentially inappropriate, use of preferred drugs and generic prescribing and assessment of the need for commonly prescribed preventive medicines. This research aims to increase the quality of prescribing in primary care, support the potential for interprofessional collaborations in primary care and contribute cost savings to the payer (i.e. the HSE) and the patient. The study will assess the feasibility of implementing processes to (a) improve appropriate prescribing in primary care and reduce PIP, (b) evaluate the costs associated with the intervention and (c) follow patient-reported outcome measures to determine changes in patient wellbeing and satisfaction with the intervention.

Most of the older people in Ireland are living independently in the community, and approximately 13% of people living in Ireland are aged ≥ 65 years [30]. This has implications for the prevalence of multimorbidity, frailty, polypharmacy and other symptoms associated with ageing, though multimorbidity and polypharmacy are increasingly an issue across the age spectrum [31]. A pharmacist, based in general practice, is ideally situated to support the management of these complex patients. An intervention such as this could enhance collaboration between healthcare professionals, improve medicines management for individual patients and improve health outcomes through safer and more effective medicines management [32].

Despite the relatively widespread implementation of practice-based pharmacists in the UK, there is limited evidence on the clinical- and cost-effectiveness of this type of intervention to date. Moreover, there are significant differences between the general practice settings that exist in the UK (publicly funded) and other countries such as Ireland (mixed publicly and privately funded). Therefore, this study has potential national and international impact and importance. Furthermore, this research has the capacity to provide a safer and higher quality service to patients by targeting outcomes at an early stage in primary care and introducing medicines management interventions that will have an additive effect on services and programmes both locally and nationally.

### Study status

At the time of this publication, four general practices and three clinical pharmacists have been recruited. Pre-intervention interviews have been undertaken with the GPs and pharmacists involved. The intervention period has started in each of the four practices, and practice-level data collection is on-going. PROM patient identification and recruitment and PROM data collection are underway.

## Abbreviations

A&E: Accident and emergency
ADR: Adverse drug reaction
CPCR: Centre for Primary Care Research
GP: General practitioner
GPP: General practice pharmacist
HRB: Health Research Board
HSE: Health Service Executive
MMP: Medicines Management Programme
MRC: Medical Research Council
NHS: National Health Service
PIP: Potentially inappropriate prescribing
PROM: Patient Reported Outcome Measures
RCT: Randomised controlled trial
SD: Standard deviation
UK: United Kingdom
